# Exploring efficacy and safety of oral Pirfenidone for progressive, non-IPF lung fibrosis (RELIEF) - a randomized, double-blind, placebo-controlled, parallel group, multi-center, phase II trial

**DOI:** 10.1186/s12890-017-0462-y

**Published:** 2017-09-06

**Authors:** Jürgen Behr, Petra Neuser, Antje Prasse, Michael Kreuter, Klaus Rabe, Carmen Schade-Brittinger, Jasmin Wagner, Andreas Günther

**Affiliations:** 1 Department of Internal Medicine V, Comprehensive Pneumology Center, University of Munich (LMU) and Asklepios Fachkliniken München-Gauting, Marchioninistr. 15, 81377 Munich, Member of the German Center for Lung Research (DZL) Germany; 2 Coordinating Center for Clinical Trials, Philipps University of Marburg, Marburg, Germany; 3 Department of Respiratory Medicine, Hannover Medical School, Hannover, Germany; 4 Department of Pneumology and Respiratory Critical Care Medicine, Thoraxklinik, University of Heidelberg, Heidelberg, Germany; 5 Lungenclinic Grosshansdorf and University of Kiel, Kiel, Germany; 6 Department of Internal Medicine II, University of Giessen-Marburg Lung Center, Justus-Liebig University Giessen, Giessen, Germany; 7 AGAPLESION Lung Clinic Waldhof-Elgershausen, Greifenstein, Germany

## Abstract

**Background:**

Pirfenidone is currently approved in the EU for the treatment of mild to moderate idiopathic pulmonary fibrosis (IPF) and offers a beneficial risk-benefit profile. However, there are several other, progressive fibrotic lung diseases, in which conventional anti-inflammatory therapy is not sufficiently effective and antifibrotic therapies may offer a novel treatment option.

**Methods/Design:**

We designed a study protocol for inclusion of patients with progressive fibrotic lung disease despite conventional anti-inflammatory therapy (EudraCT 2014–000861-32). The study population comprises patients with collagen-vascular disease-associated lung fibrosis (CVD-LF), fibrotic non-specific interstitial pneumonia (fNSIP), chronic hypersensitivity pneumonitis (cHP), and asbestos-related lung fibrosis (ALF). Disease progression needs to be proven by slope calculation of at least three Forced Vital Capacity (FVC) values obtained within 6–24 months prior to inclusion, documenting an annualized decline in percent predicted FVC of 5% (absolute) or more despite appropriate conventional therapy. Absolute change in percent predicted FVC from baseline - analyzed using a rank analysis of covariance (ANCOVA) model - will serve as efficacy-related primary study endpoint.

**Discussion:**

There is an urgent unmet clinical need for effective therapies for patients with a progressive fibrotic lung disease other than IPF. The current study protocol is unique with respect to selecting patients with different disease entities of lung fibrosis which have, however, essential pathophysiological characteristics in common. Moreover, by selecting patients with evidence of disease progression despite conventional therapy, the protocol ensures that a cohort of interstitial lung disease (ILD) patients with a high unmet medical need is targeted and it may allow a sufficiently high event rate for evaluation of treatment responses.

**Trial registration:**

DRKS00009822 (registration date: January 13th 2016).

## Background

In the years 2011–2015, the anti-fibrotic drug pirfenidone (inhibitor of TGF-β, TNF α and of PDGF) and the triple kinase inhibitor nintedanib (inhibitor of PDGF, VEGF, and FGF) have both been approved for treatment of Idiopathic Pulmonary Fibrosis (IPF) in the European Union as well as in the United States. IPF is the most frequent and aggressive diffuse parenchymal lung disease (DPLD) in the western world. In well-designed clinical phase III trials, both drugs have been demonstrated to reduce the annual loss of FVC by approximately 50% and to yield a significant reduction of all-cause mortality after one year for pirfenidone [1–4]. Of note, there are several DPLDs which share pathomechanistic principles, e.g. disturbed epithelial-mesenchymal crosstalk, radiographic/histological characteristics like reticulation, traction bronchiectasis and honeycombing in variable combinations, as well as the progressive worsening and the fatal outcome with IPF and, therefore, represent important differential diagnoses and areas of great unmet medical need. Among these are lung fibrosis in association to collagen/vascular diseases (CVD-LF), fibrotic non-specific interstitial pneumonia (fNSIP), chronic hypersensitivity pneumonitis (cHP), and asbestos-induced lung fibrosis (ALF) [5–12]. In these entities, immunosuppressive therapy represents the standard clinical treatment option, albeit frequently ineffective and potentially harmful. In case of ALF, no pharmacological treatment is available at all. Therefore, a novel treatment option is highly needed for these most severely impaired patients.

We hypothesized that, based on the pathophysiological and clinical similarities between IPF and the mentioned four types of non-IPF lung fibrosis, pirfenidone should be a viable treatment option especially in patients in whom conventional anti-inflammatory therapy failed. In particular, we aim to assess whether treatment with pirfenidone results in attenuation of the decline in lung function and exercise capacity in these patients, similarly to what has been observed in IPF. We will therefore conduct an exploratory efficacy and safety study of oral pirfenidone for progressive, non-IPF lung fibrosis (RELIEF in LUNG FIBROSIS) in CVD-LF, fNSIP, cHP, and ALF.

### Funding and ethics

This protocol is funded by the German Center for Lung Research (Bundesministerium für Bildung und Forschung, BMBF) and co-funded by Roche Pharmaceuticals. It has been approved by the ethics committee of the Ludwig-Maximilians University of Munich, Germany. Written informed consent is obtained from every patient enrolled.

### CVD-lf

Lung fibrosis is a common feature in CVD: it can be found in up to 80% of patients with progressive systemic sclerosis (PSS) and in 20–50% of patients with rheumatoid arthritis (RA) and it is not rarely observed in patients with systemic lupus erythematosus, dermatomyositis/polymyositis and Sjögren’s syndrome [5]. Musculoskeletal pain, weakness, fatigue, fever, joint pain or swelling, photosensitivity, Raynaud’s phenomenon, pleuritis, dry eyes, or dry mouth in the setting of lung fibrosis are important hints for the existence of CVD-LF, but these symptoms may appear later, after lung fibrosis has already been diagnosed. Detection of auto-antibodies such as antinuclear antibodies, antibodies against extractable nuclear antigens or of rheumatoid factor/ anti citrullinated peptide antibodies (ACPA) may be helpful to early disclose the link between lung fibrosis and a systemic autoimmune disease. The most common form of pulmonary involvement is the nonspecific interstitial pneumonia histopathologic (NSIP) pattern, but a UIP pattern is also regularly encountered [6]. Administration of immunosuppressant drugs may be of help in some forms of CVD-LF (e.g. lung fibrosis associated with Sjögren’s syndrome), but usually fails to exert beneficial effects and disease control in the long term (e.g. in PSS). Importantly, pulmonary involvement usually is the key prognostic factor in these CVD and progression of lung fibrosis is associated with worse outcome.

### Idiopathic fibrotic NSIP

Originally designed as provisional entity, idiopathic NSIP is meanwhile regarded as a separate and clearly distinguishable subgroup within the idiopathic interstitial pneumonias (IIPs) [7]. NSIP seems to come along in two peculiarities, one being the cellular form, with extensive, general lymphoplasmacellular infiltrations of the septae, resulting in septal thickening, but not that much fibrosis, the other one being fibrotic NSIP, being characterized by deposition of collagen in the septae and a more variable extent of inflammatory reaction. Typically, however, the alveolar structure is maintained and complex fibrosis, such as honeycombing as observed in UIP, is absent. Although the clinical picture is related to IPF (patients are only slightly younger and more frequently female), with coughing and exertional dyspnea representing the key complaints, the HRCT is clearly different, showing extensive ground glass opacities in cellular NSIP and reticular changes in fibrotic NSIP, usually not following a strict centripetal gradient and being also present frequently in upper lobes [8]. Patients with cellular NSIP are usually well responding to a corticosteroid or combined immunosuppressive therapy, their life-expectancy is probably not reduced. In contrast, patients with fibrotic NSIP do show a much more progressive phenotype of the disease, with a varying degree of responsiveness to immunosuppressive treatment [7]. In case such therapy fails, progressive fibrosis occurs and will be prognostically relevant.

### cHP

cHP is a type III/IV based allergic reaction, with development of specific (so called precipitating) IgG antibodies against inhaled organic antigens [9]. The spectrum of the causative antigens is broad, covering a multitude of antigens associated with occupational (e.g. actinomyces thermophilus in case of the farmer lung) or leisure (e.g. pigeon droppings and feathers in case of pigeon breeders lung) activities. In the more acute stage, respiratory (dyspnea, coughing) and more general (fever, headaches) symptoms start some hours after antigen exposure. During this acute episode, a primarily lymphocytic inflammatory reaction takes place in the lung parenchyma, results in the formation of granuloma and is usually associated with a CD8 dominant lymphocytic reaction in the bronchoalveolar lavage fluid (BALF; CD4/CD8 < 1) [9]. During this phase, the clinical picture and the histopathological (NSIP-like pattern, lymphoplasmacellular infiltrations, granuloma formation), radiographic (nodular pattern and ground glass primarily in upper lobes), and functional (hypoxemia, restrictive and/or obstructive ventilation pattern) abnormalities are fully reversible upon antigen cessation. In case of a permanent and / or prolonged antigen exposure (either due to unawareness or unavoidable contact to a known antigen), the above mentioned temporal and/or spatial relationship between antigen exposure and symptoms is lost, and there is a variably rapid and progressive decline in lung function and exercise capacity. In this phase, considerable percentages of neutrophils are found in BALF, granuloma may be missing in the histopathology and a usual interstitial pneumonia (UIP) pattern may be observed [10]. In high-resolution computed tomography (HRCT), fibrotic changes may mimic the classical UIP pattern of IPF, but with peribronchiolar and upper lobe predominance, and may result in a more complex picture, making the correct diagnosis quite difficult [11]. Also, in contrast to the primarily inflammatory triggered early phase, an increasing number of reports suggest that epithelial injury and apoptosis, a key feature of IPF, play a significant role in cHP, too [12]. Immunosuppressive treatment with corticosteroids is known do ameliorate acute episodes of HP, but has questionable effects on chronic HP which may progress with and without persistent antigen exposure [11].

### Alf

Despite the prohibition of the use of asbestos in the early ‘90s, asbestos-related lung diseases are on the peak nowadays, illustrating the long latency observed in these diseases. Whereas pleural asbestosis is easily diagnosed on the basis of a corresponding patient’s history and the typical radiographic appearance in HRCT, diagnosing pulmonary involvement is more complex. If pleural changes typical of asbestos-induced lung diseases are present, any kind of interstitial lung disease should be regarded asbestos-induced, unless a known other cause is identified. In absence of prototypical pleural changes, however, the association between radiographic and histopathological findings of pulmonary fibrosis, sometimes even resembling a typical UIP pattern, and asbestos can only be made on the basis of a thorough, extensive patient history, with a focus on the professional activities [13]. Typically, the disease is progressive in nature, with a varying rate of progression. There is no established treatment for these patients and steroids or immunosuppressants are typically not regarded as effective. Apart from the progressive nature of lung fibrosis, patients with ALF or pleural asbestosis are facing the challenge of lung cancer or mesothelioma development, which then largely determines the prognosis. Interestingly, more recent reports also indicate ongoing epithelial injury in asbestos-induced lung fibrosis [14].

### Efficacy and safety of pirfenidone in clinical IPF trials

Clinical safety and efficacy data on oral pirfenidone for the treatment of patients with IPF and PF include the following: Three published journal articles on clinical studies evaluating oral pirfenidone for the treatment of patients with IPF [15–17]; one controlled Phase II trial was published in abstract form but not published as a journal article in the medical literature: the study, PIPF-001, was initiated by Marnac, the original Sponsor, and completed by InterMune [18]; one study published by the National Institutes of Health (NIH) using pirfenidone in patients with PF associated with Hermansky-Pudlak Syndrome (HPS) [19]; one controlled, prospective, randomized clinical trial evaluating oral pirfenidone in Japanese IPF patients [20]; two controlled, prospective, randomized clinical Phase III trials evaluating oral pirfenidone in IPF patients with change in FVC as primary endpoint (CAPACITY program, PIPF 004 and PIPF 006) [1]; one pivotal phase III clinical trial (ASCEND) confirming the beneficial effect of pirfenidone on decline of FVC, decline of 6MWD, and a significant effect on overall mortality [2]. There is a pooled analysis of the three randomized clinical trials [1, 2] available [3], as well as a meta-analysis summarizing some of the clinical observations made with pirfenidone in patients with IPF [21].

On the basis of these studies, pirfenidone was authorized as treatment for mild to moderate IPF in the EU in the year 2011 and in the US after completion of the confirmatory ASCEND trial in 2014. In total, more than 1650 volunteers and patients received at least one dose of pirfenidone in clinical trials. Pirfenidone was administered orally TID at total doses ranging from 1197 mg/d to 3600 mg/d. The incidence of adverse events (AEs) in clinical studies was high (approximately 80–90%) but they were comparable in the pirfenidone and placebo arms and in general classified as mild to moderate. In all clinical trials, the side effect profile of pirfenidone was similar, with gastrointestinal malaise, anorexia, weight loss being most frequent, followed by skin rash and photosensitivity requiring permanent use of sun blockers. Overall, the side effects were tolerable for the vast majority of IPF patients and only led in approximately 5% to treatment discontinuation.

### Rationale for the RELIEF study

As outlined above, IPF is not the only fibrotic lung disease being characterized by a UIP pattern and by primarily fibrotic (reticular) changes in HRCT. Similar changes may be encountered in CVD-LF, cHP, and ALF. Although distinct in terms of histopathology and HRCT, fNSIP may act in a clinically similar manner as compared to IPF. Therefore, if immunosuppressive therapy, usually administered in CVD-LF, fNSIP, and cHP fails to stop the progression of the disease, the life-expectancy of patients with these forms of diffuse parenchymal lung disease is likewise limited by the progressive nature of the lung fibrosis. Against this background it appears more than reasonable to speculate that pirfenidone, a drug with a yet not completely known molecular action profile, but profound anti-fibrotic and even anti-inflammatory activities, may not only be effective in IPF, but also in other, progressive forms of lung fibrosis, where anti-inflammatory treatment modalities fail to exert arrest of progression and/or improvement.

### Aims of the study

The purpose of the RELIEF study is to assess the safety and efficacy of treatment with pirfenidone (2403 mg/d) compared to placebo in patients with CVD-LF, fNSIP, cHP, and ALF.

## Methods/Design

RELIEF is a prospective, randomized (1:1), placebo-controlled clinical trial enrolling patients with progressive fibrotic lung disease due to CVD-LF, fNSIP, cHP or ALF, despite conventional anti-inflammatory therapy. The progressive character of the underlying fibrotic lung disease has to be objectively documented by at least three pulmonary function tests within 6 to 24 months prior to enrollment demonstrating an annualized percent predicted FVC decline of ≥5% (absolute). The change in lung function is determined by employing the DZL-FVC slope calculator, a tool available on the German Center for Lung Research DZL website (http://www.dzl.de/index.php/de/forschung/tools/fvc-slope-calculator).

### Study endpoints

Efficacy will be assessed by the absolute change in percent predicted FVC from baseline to week 48 using a rank analysis of covariance (ANCOVA) model.

#### A number of key secondary endpoints were defined:

1. Time to disease worsening, defined as time to clinical deterioration, lung fibrosis-related death, lung transplant or respiratory hospitalization, whichever comes first

For the purpose of this study, clinical deterioration is defined as follows:

1. Clinically worsening of dyspnea within 4 weeks and
2. New or worsening radiographic abnormalities on chest x-ray or HRCT and
3. Objective worsening of PFT or gas exchange defined by at least one of the following criteria
  - Initiation of LTOT or increase of oxygen supplementation of existing LTOT by ≥1 l/min to maintain resting oxygen saturation (SaO_2_) ≥ 90%.
  - Drop in FVC by >10% as compared to last measurement
  - Drop in DLCO by >15% as compared to last measurement
  - Drop in 6MWD by 20% as compared to last measurement
4. Progression-free survival, defined as time to the first occurrence of either of the following (as compared to the patient’s baseline):
  - ≥ 10% absolute decline in percent predicted FVC
  - ≥ 15% absolute decline in percent predicted Hb-corrected DLCO
  - Death of the patient
5. Categorical assessment of relative change from baseline to week 48 in percent predicted FVC.
6. Change from baseline to week 48 in SGRQ and EQ-5D.
7. Change from baseline to week 48 in the percent predicted Hb-corrected DLCO/TLCO.
8. Change from baseline to week 48 in the worst oxygen saturation by pulse oximetry (SpO2) measurement observed during the 6MWT.
9. Change from baseline to week 48 in distance walked in the 6MWT.
10. Assessment of safety including overall mortality.

### Study population

Symptomatic patients at the age of 18–80 years with a confident diagnosis (at least 9 months before randomization) of progressive, non-IPF lung fibrosis due to ALF, CVD-LF, cHP or fNSIP according to the diagnostic criteria outlined in Table 1 are eligible for this study. In addition, disease progression despite previous or concomitant treatment (CVD-LF, chronic HP, fNSIP) or without specific therapy (ALF) must be documented by calculating the slope of a set of at least three previous FVC measurements within at least six to a maximum of 24 months before screening, showing an (eventually extrapolated) FVC decline of at least 5 % (abs. Pred.) per year.

**Table 1:** Diagnostic Criteria of RELIEF Patient Categories (all to be fulfilled within each category)

Lung fibrosis associated with collagen / vascular diseases
• Diagnosis of progressive systemic sclerosis (PSS), rheumatoid arthritis (RA), Sjörgen’s syndrome, polymyositis/dermatomyositis on the basis of extrapulmonary symptoms and corresponding proof of auto-antibodies
• Reticular changes in HRCT and restrictive lung function pattern
• Absence of an alternative explanation for fibrotic lung disease
Fibrotic NSIP
• Histological diagnosis of a fibrotic NSIP pattern by open lung biopsy or cryobiopsy
• HRCT consistent with fibrotic NSIP
• Restrictive lung function pattern
• Absence of an alternative explanation for fibrotic lung disease, especially no clinical suspicion of CVD
Chronic Hypersensitivity Pneumonitis
• Previous or current respiratory symptoms (dyspnea, coughing) with a temporal or spatial relation to a causative antigen exposure
• Proof of precipitating antibody and/or lymphocytic alveolitis (>30%)
• HRCT consistent with chronic HP
• Restrictive lung function pattern
• Absence of an alternative explanation for fibrotic lung disease
Asbestos-induced lung fibrosis
• Existence of asbestos-specific pleural changes in HRCT (pleural plaques)
• Reticular changes in HRCT and restrictive lung function pattern
• History of asbestos exposure
• Absence of an alternative explanation for fibrotic lung disease
• Absence of extensive pleural plaques and/or effusion

Additionally, the following inclusion criteria were defined:

1. Women of childbearing capacity are required to have a negative serum pregnancy test before treatment and must agree to maintain highly effective methods of contraception by practicing abstinence or by using at least two methods of birth control from the date of consent through the end of the study. If abstinence is not practiced, then one of the two methods of birth control should be an oral contraceptive (e.g., oral contraception and a spermicide)
2. Percent predicted FVC ≥ 40%, but <90% at the Screening Visit (before randomization)
3. Percent predicted, hemoglobin (Hb)-corrected carbon monoxide diffusing capacity/carbon monoxide transfer capacity (DLCO) ≥ 25%, but <75% at the Screening Visit
4. Distance walked ≥150 m, with O_2_ saturation > 83% on ≤6 L/min of O_2_
5. Able to understand and sign a written informed consent form
6. Able to understand the importance of adherence to study treatment and the study protocol, including the concomitant medication restrictions throughout the study period
7. Patients being considered or on the waiting list for lung transplantation are eligible for participation only if their estimated waiting time is longer than the study period. At the time these patients are placed on the list the date and reason for this placement and the Lung Allocation Score (LAS) should be collected

### Exclusion criteria were defined as follows

#### Disease-Related Exclusions:

1. Not a suitable candidate for enrollment or unlikely to comply with the requirements of this study, in the opinion of the investigator
2. May not survive the study period, in the opinion of the investigator
3. Premature withdrawal from a randomized clinical trial in the previous 2 years for any reason other than sponsor decision or current participation in a clinical drug trial
4. Obvious additional obstructive lung disease, as evident from a) forced expiratory volume in the first second (FEV1)/FVC ratio < 0.7 after administration of a bronchodilator at Screening Visit, OR b) bronchodilator response defined by an increase of ≥12% and an increase of ≥200 mL in the FEV1 after bronchodilator use compared to the value seen before bronchodilator at the Screening Visit, OR c) residual volume (RV) > 140% of predicted (before administration of bronchodilator)
5. Other explanation for interstitial lung disease, including but not limited to radiation, sarcoidosis, bronchiolitis obliterans organizing pneumonia, human immunodeficiency virus (HIV), viral hepatitis and cancer
6. Clinical evidence of active infection, including but not limited to bronchitis, pneumonia, sinusitis, urinary tract infection, or cellulitis
7. In the clinical opinion of the investigator, the patient is expected to need and be eligible for a lung transplant within 52 weeks after randomization
8. Unable to undergo pulmonary function testing

#### Medical Exclusions:

9. Any history of malignancy likely to result in death or significant disability or likely to require significant medical or surgical intervention within the next 2 years. This does not include minor surgical procedures for localized carcinoma (e.g., basal cell carcinoma).
10. Any condition other than lung fibrosis which, in the opinion of the investigator, is likely to result in the death of the patient within the next 2 years.
11. History of advanced cirrhosis or clinically significant liver disease.
12. History of unstable or deteriorating cardiac or pulmonary disease (other than IPF) within the previous 6 months, including but not limited to the following:
  - Myocardial infarction, unstable angina pectoris, coronary artery bypass surgery, or coronary angioplasty
  - Congestive heart failure requiring hospitalization
  - Uncontrolled arrhythmias
  - Asthma or chronic bronchitis requiring hospitalization in the last 6 months
13. Any condition, which, in the opinion of the investigator, might be significantly exacerbated by the known side effects associated with the administration of pirfenidone.
14. Poorly controlled diabetes (defined by glycosylated hemoglobin [HbA1C] >10%).
15. Pregnancy or lactation.
16. History of alcohol or substance abuse in the past 2 years.
17. History of any condition or habit associated with altered consciousness and a risk of aspiration in the past 2 years.
18. Family or personal history of long QT Syndrome.
19. Treatment with either one of the following medications: fluvoxamine (any time), any investigational drug, PH-specific mediation unless initiated >6 months ago and kept stable thereafter and throughout study period.
20. Severe pulmonary hypertension with PVR values >900 dyn.
21. Smoking.

#### Laboratory Exclusions:

22. Any of the following liver function test criteria above specified limits: Total bilirubin >2.5 upper limit of normal (ULN); aspartate or alanine aminotransferase (AST/SGOT or ALT/SGPT) >2.5 ULN; alkaline phosphatase >2.5 ULN

#### Concomitant Therapy Exclusions:

23. Severe liver failure.
24. Prior use of pirfenidone.
25. Hypersensitivity/allergy against pirfenidone or any component of the investigational medicinal product (IMP).
26. History of angioedema in relation to prior use of pirfenidone.
27. Severe renal failure defined as GFR < 30 ml/min and/or need for dialysis.
28. Concomitant therapy with potential interaction to study drug according to SmPC.
29. Patients are excluded if more than 15 mg prednisolon equivalent are applied per day or if a pre-existent steroid and/or immunosuppressant therapy has been modified within the last 3 months and/or if such therapy would be needed to be changed during the study period. In that case, dosing has to be adjusted and patient must be re-checked for eligibility 3 months after the last dose adjustment.

### Concomitant therapy

Drugs that are considered necessary for the patient’s welfare may be given at the discretion of the investigator with respect to the potential interactions according to Summary of Product Characteristics (SMPC). However, careful monitoring for new symptoms by the patient is vital in this instance. Information on concomitant medications will be collected until the final Follow-Up Visit. For patients who withdraw from study, data on concomitant medications will be collected until the later of the following two time points:

1. Time of consent withdrawal.
2. 28 days after last dose of study treatment.

With the exception of clinical deterioration (for definition see study endpoints) due to disease.

progression therapies listed below are not permitted during the study:

- Any investigational therapies (i.e., agents that are not approved by local regulatory agencies, including N-acetyl-cysteine (NAC) at a dose >600 mg/d)

- Any interferon therapy, including IFN-γ 1b

- Any cytokine modulators (including but not limited to etanercept and infliximab)

- Any other therapy that has been investigated or used off-label as a treatment for lung fibrosis including but not limited to d-penicillamine, colchicine, bosentan, imatinib mesylate, and cyclosporine A, rituximab. If patients receive any of these excluded therapies while on study, it will be recorded in the e-CRF and will be considered as a protocol violation.

In case of a clinical deterioration due to disease progression, pulse-dose steroids may be used for a period of up to 14 days, as well as any of the above mentioned and other “rescue” therapies such as plasma separation. This will be entirely left to the discretion of the investigator and have to be documented in the e-CRF.

## Statistical considerations

### Hypotheses

Hierarchical testing will be applied in the final analysis to test the treatment effect in different groups with regard to diagnosis (see Table 1 for diagnostic categories). The hypothesis H_0_: “no difference in absolute change in percent predicted FVC from baseline to week 48 between the pirfenidone and placebo group” will be tested against the alternative H_1_: “difference in absolute change in percent predicted FVC from baseline to week 48 between the pirfenidone and placebo group”. The order of hypothesis testing is defined as follows:

1. H_0_ against H_1_ in the overall group
2. H_0_ against H_1_ in the group in which the diagnosis group with the smallest number of patients is excluded
3. H_0_ against H_1_ in the group in which the diagnosis group with the second smallest number of patients is excluded
4. H_0_ against H_1_ in the diagnosis group which has the highest number of patients
5. H_0_ against H_1_ in the diagnosis group which has the second highest number of patients
6. H_0_ against H_1_ in the diagnosis group which has the third highest number of patients
7. H_0_ against H_1_ in the diagnosis group which has the smallest number of patients

Hypotheses are tested consecutively until the *p*-value of a hypothesis exceeds the 5% significance level, thereby controlling the familywise error rate (FWER) at the 5% level.

### Analysis population

The intention-to-treat (ITT) population is defined as all randomized patients. All analyses of outcome parameters will be based on the intention-to-treat population.

The per protocol (PP) population comprises all patients who had no major protocol deviation with respect to the primary endpoint. The analysis of the primary endpoint will also be provided for the per protocol population.

The as treated population is defined as all included patients who received at least one dose of the investigational medicinal product (IMP). Analysis of safety will be based on the as treated population, i. e. patients will be analyzed according to the treatment they actually received.

### Sample size

The sample size and power calculations are based on the analysis of the primary efficacy endpoint in the overall group: absolute change in percent predicted FVC from baseline to week 48. It is assumed that the primary efficacy endpoint is not normally distributed and that its probability distributions are identical in both treatment populations, except for location. Assuming furthermore a similar treatment effect in all clinical diagnosis groups (see Table 1 for diagnostic categories), the two-sample Mann-Whitney U Test for independent samples is used to test for a difference in median. For a group sequential design according to O’Brien/Fleming with one interim analysis, a two-sided significance level of 5% and a power of 80%, 187 patients per arm are needed to detect an effect size of 0.3 between the two treatment groups across all indications. The magnitude of the effect size was based on the observed rank effects (i.e. effect in a non-parametric analysis) of pirfenidone versus placebo in the CAPACITY study at 1 year (48 weeks) corresponding to an effect size of 30% after scaling back. In the pooled CAPACITY studies the effect size was around 40%. The conservative choice of the effect size allows for a dropout rate like in CAPACITY [1].

Sample size calculation in ADDPLAN 6.0 is only available for group sequential designs based on t tests. Therefore, the results were multiplied by the factor π/3 for nonparametric adjustment as proposed by Al-Sunduqchi and Guenther (1990) (Determining the Appropriate Sample Size for Inferences Based on the Wilcoxon Statistics. Ph.D. dissertation under the direction of William C. Guenther, Dept. of Statistics, University of Wyoming, Laramie, Wyoming) and implemented in PASS 2008 [22].

### Interim analysis

There is one planned interim analysis for efficacy. An interim analysis will be performed after completion of treatment in 224 patients which is expected to occur at about two and a half years after start of accrual. The significance level in the interim analysis is 0.0101 and the power for a positive result in the interim analysis is about 35% if the true effect size equals 0.3.

### Primary endpoint

The primary efficacy analysis will be to investigate the treatment effect on the absolute change in percent predicted FVC from baseline in the pirfenidone and placebo group with regard to each hypothesis tested. As the primary efficacy endpoint is assumed to be not normally distributed, data will be analyzed using a rank analysis of covariance (ANCOVA) model with a classification effect for treatment and diagnostic category and baseline FVC as a covariate. The null hypothesis is tested against the alternative hypothesis at a two-sided significance level of 5%. The treatment effect will be tested using the Cochran-Mantel-Haenszel mean score statistic, median treatment differences will be estimated according to Hodges-Lehmann. The applied methods are based on a location-shift model assuming that the metric distributions of the absolute change in percent predicted FVC are the same, except for location. Furthermore, the primary efficacy endpoint will be analyzed by factorial ANCOVA with a classification effect for treatment and diagnostic category (with and without an interaction term for treatment and diagnostic category) and baseline FVC as a covariate.

### Handling of missing values in rank ANCOVA

Patients with missing values due to death will be assigned the worst ranks according to the time from randomization until death where the shortest time until death corresponds to the worst rank.

A missing value at Visit X in Patient A who is alive at that time will be imputed as follows. For patients without any missing values from baseline up to Visit X the sum of squared differences (SSD) between each patient and Patient A is calculated across all values from baseline up to one visit before Visit X. The three patients with the smallest SSD will be taken into account for imputing the missing value for Patient A at Visit X by taking the average value of the three patients at that visit.

### Secondary endpoints

Secondary outcomes will be analyzed by using appropriate statistical methods, e.g. Kaplan-Meier estimates and rank ANCOVA. Additional analyses will be described in detail in a statistical analysis plan (SAP) before data base closure.

### Safety endpoints

Safety data will be summarized by using descriptive statistical methods.

## Discussion

The study is unique for several reasons. First, it allows to enroll patients with different forms of interstitial lung disease. Second, a common progressive fibrotic phenotype of ILD is defined by radiomorphology on high-resolution thin section computed tomography and, more importantly, by at least three spirometric measurements within 6 to 24 months, which show an annual decline in percent predicted FVC of at least 5% (absolute). Third, study medication (i.e. pirfenidone or placebo) is used on top of existing anti-inflammatory medication, which has to be stable for at least three months. If progressive fibrosis is the common pathophysiological pathway in these diseases, there is good chance that antifibrotic effects of pirfenidone ameliorate disease progression in this group of diseases.

### Trial status

Initiation of the participating sixteen centers throughout Germany started in April 2016. In March 2017 four additional centers were activated and by May 3rd 2017 a total of 74 patients have been randomized.

## Key messages

Fibrotic interstitial lung disease other than IPF comprises a variety of different diseases in which current conventional anti-inflammatory therapies are frequently ineffective.

There is an urgent unmet clinical need for effective therapies for interstitial lung diseases with a progressive fibrotic phenotype other than IPF.

Pirfenidone has shown a favorable benefit-risk profile in IPF and therefore appears highly likely to be effective also in other fibrotic lung diseases.

The RELIEF protocol allows enrollment of patients with four selected disease entities and evidence of disease progression despite anti-inflammatory therapy as a Phase II study for the use of antifibrotic drugs in these diseases.
